# Genomic architecture of fetal central nervous system anomalies using whole-genome sequencing

**DOI:** 10.1038/s41525-022-00301-4

**Published:** 2022-05-13

**Authors:** Ying Yang, Sheng Zhao, Guoqiang Sun, Fang Chen, Tongda Zhang, Jieping Song, Wenzhong Yang, Lin Wang, Nianji Zhan, Xiaohong Yang, Xia Zhu, Bin Rao, Zhenzhen Yin, Jing Zhou, Haisheng Yan, Yushan Huang, Jingyu Ye, Hui Huang, Chen Cheng, Shida Zhu, Jian Guo, Xun Xu, Xinlin Chen

**Affiliations:** 1 BGI-Shenzhen, Shenzhen, 518083 China; 2 Maternal and Child Health Hospital of Hubei Province, Hubei, 430070 China

**Keywords:** Medical genomics, Molecular medicine

## Abstract

Structural anomalies of the central nervous system (CNS) are one of the most common fetal anomalies found during prenatal imaging. However, the genomic architecture of prenatal imaging phenotypes has not yet been systematically studied in a large cohort. Patients diagnosed with fetal CNS anomalies were identified from medical records and images. Fetal samples were subjected to low-pass and deep whole-genome sequencing (WGS) for aneuploid, copy number variation (CNV), single-nucleotide variant (SNV, including insertions/deletions (indels)), and small CNV identification. The clinical significance of variants was interpreted based on a candidate gene list constructed from ultrasound phenotypes. In total, 162 fetuses with 11 common CNS anomalies were enrolled in this study. Primary diagnosis was achieved in 62 cases, with an overall diagnostic rate of 38.3%. Causative variants included 18 aneuploids, 17 CNVs, three small CNVs, and 24 SNVs. Among the 24 SNVs, 15 were novel mutations not reported previously. Furthermore, 29 key genes of diagnostic variants and critical genes of pathogenic CNVs were identified, including five recurrent genes: i.e., *TUBA1A*, *KAT6B*, *CC2D2A*, *PDHA1*, and *NF1*. Diagnostic variants were present in 34 (70.8%) out of 48 fetuses with both CNS and non-CNS malformations, and in 28 (24.6%) out of 114 fetuses with CNS anomalies only. Hypoplasia of the cerebellum (including the cerebellar vermis) and holoprosencephaly had the highest primary diagnosis yields (>70%), while only four (11.8%) out of 34 neural tube defects achieved genetic diagnosis. Compared with the control group, rare singleton loss-of-function variants (SLoFVs) were significantly accumulated in the patient cohort.

## Introduction

Fetal central nervous system (CNS) anomalies are among the most common congenital abnormalities. For example, the prevalence of neural tube defects (NTDs) is estimated to be 18.6/10,000 live births globally^1^. However, the incidence of intracranial malformations with an intact neural tube is not certain as most escape detection prenatally or at birth and only manifest in later life^2^. Therefore, screening and diagnosing CNS anomalies as early as possible is essential to provide critical information for clinical decisions. Ultrasonography is the most important modality for evaluating fetal CNS defects^3^. Fetal magnetic resonance imaging (MRI) can also provide additional information for fetal CNS assessment^4^. However, prenatal diagnosis of CNS anomalies remains challenging due to ongoing growth and fetal brain development^5^.

Genomic variations are important etiologies of fetal anomalies. Karyotyping and chromosomal microarray analysis (CMA) are effective at diagnosing chromosomal anomalies and copy number variations (CNVs)^6^. In current clinical practice, exome sequencing (ES) is usually applied after uninformative CMA results to detect single-gene mutations on exons^7,8^. This strategy may be economical, but it overlooks the fact that pathogenic genetic variation can differ considerably among congenital diseases. Furthermore, time is very limited for accurate fetal diagnosis. Therefore, a one-for-all genetic testing modality is necessary^9^. Whole-genome sequencing (WGS) with an average depth of 30–40X can detect CNVs, single-gene mutations, and structural variations such as intragenic CNVs and intron mutations. Various studies have called for an expansion in diagnostic utility for infants and children for a wide spectrum of disorders^10,11^. However, although the cost of WGS is decreasing rapidly, it is still expensive compared to CMA and ES. Therefore, the genomic background of imaging phenotypes could achieve a balance between cost and efficiency. Although hundreds of genetic syndromes involving the CNS have been studied, their prenatal imaging manifestations remain poorly described. Furthermore, genomic studies of large cohorts with fetal CNS malformations are lacking. In this study, the genomic landscapes of 162 unselected fetuses were explored to demonstrate the distribution of genomic variations and their characteristics in major types of ultrasonographic-detected fetal CNS anomalies, including multifactorial diseases such as NTDs and hydrocephalus, to gain a better understanding of CNS anomalies. The diagnostic power of WGS for fetal CNS ultrasound anomalies was evaluated, thus providing a solid foundation for flexible choice in prenatal genetic screening and diagnosis.

## Results

### Demographic characteristics of cohort and prenatal imaging features

Detailed clinical information such as pregnancy history, gestation, prenatal ultrasound phenotypes, and post-mortem examinations of all fetuses were reviewed and are listed in Supplementary Table 1. The median maternal age of the cohort was 27. No statistical differences in age median or distribution were detected between the cohort and Chinese women who gave birth in 2015 (determined from survey data from the National Bureau of Statistics of China)^12^. The chromosomal sex ratio of the fetuses was 1:1 (81/81), suggesting no sex differences in the cohort. More than 60% of the final ultrasound diagnoses were obtained after 28 weeks. In total, 11 typical CNS anomalies were identified in the current cohort, as shown in Supplementary Fig 1. Fetuses may have had more than one type of CNS or non-CNS malformation.

Hydrocephalus and NTDs were the most frequent phenotypes in the cohort, each occurring in 34 fetuses. In addition, 114 fetuses had anomalies in the CNS only, while 48 had both CNS and non-CNS abnormalities (not including the typical facial anomalies of holoprosencephaly or clubfeet of Chiari II malformation). Autopsy and/or prenatal MRI data were available for 83 fetuses, with additional structural malformations not diagnosed by prenatal ultrasound found in eight cases. This included two cerebral cortical malformations found by MRI, i.e., periventricular heterotopia and parietal frontal dysplasia, in addition to the aplasia of the corpus callosum (ACC) and arachnoid cysts identified by prenatal ultrasound. Other findings from the autopsy included abnormalities in the fingers and toes in three fetuses, vascular malformations in two fetuses, and right iliosacral joint malformation in one fetus.

### Sequencing and genetic diagnosis by WGS

Two-stage sequencing was applied, with 26 cases subjected to low-pass WGS only and 136 cases sequenced to an average depth of 41.9 ± 0.4-fold coverage for diagnosis and further analysis. Three fetuses failed CNV calling after both low-pass and deep WGS due to turbulence of read depth across the whole genome, although SNV analysis was not affected. Following low-pass WGS, 19 aneuploids and 342 CNVs (>100 kb) were identified. Deep WGS identified an average of 3,925,317 ± 2403 single-nucleotide polymorphisms (SNPs) and 938,371 ± 2860 indels for each sample. Small CNVs (ranging from 50 bp to 100 kb) beyond the detection limit of mainstream CMA were found in 102 samples without primary diagnosis by low-pass WGS or SNV analysis. Four samples did not pass the quality control test for small CNV analysis. On average, 2866 ± 40 small CNVs were identified in each sample, including 1203 ± 18 gene-containing CNVs and 89 ± 3 exon-containing CNVs. The numbers of CNVs containing genes and exons were linearly dependent on total number of small CNVs (Supplementary Fig. 2). Only small CNVs containing exons with a frequency lower than 0.01 in our in-house database were assessed by the clinical review panel. As a result, an average of 10.8 ± 0.9 small CNVs were assessed for each sample.

**Fig. 1:**
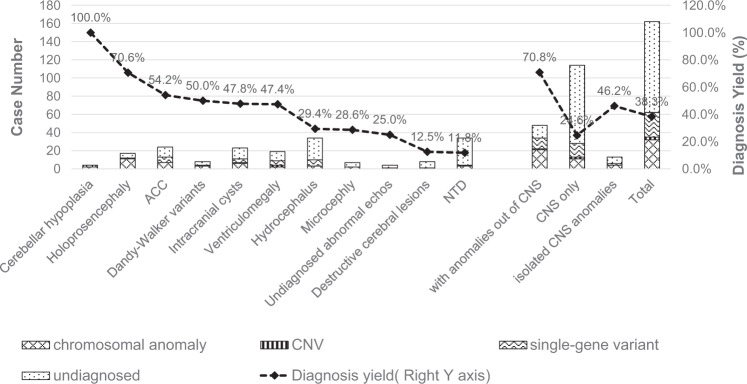
Diagnostic yields of WGS in different types of CNS anomalies and distribution of diagnostic variants. Bars indicate case numbers (left y-axis) and the dashed line shows the diagnostic rate (%) (right y-axis). Cases were repeatedly counted if more than one sonographic feature was presented. Isolated brain anomalies only included aplasia of the corpus callosum, arachnoid cyst, and ventriculomegaly, as other CNS anomalies are usually complex and involve several anatomical structures of the brain. ACC aplasia of corpus callosum.

Overall, the primary diagnosis was achieved in 62 cases (diagnosis rate of 38.3%), as shown in Fig. 1. In total, 18 aneuploids, 21 CNVs, and three small CNVs were diagnosed (Table 1). Pathogenicity scores and supporting references are listed in Supplementary Table 4. Furthermore, as shown in Table 2, 26 pathogenic or likely pathogenic SNVs were found, which may explain the fetal phenotypes. The pathogenicity criteria are listed in Supplementary Table 5. Among them, 16 are novel mutations that have not been reported previously, seven are known pathogenic variants, and three are listed in the Single‐Nucleotide Polymorphism database (dbSNP) but without clear clinical significance. A possible mosaic 47XXY was found in P1133, which showed Chiari II malformation. However, there was no evidence indicating a correlation between 47XXY and Chiari II malformation. The eight pathogenic or likely pathogenic variants classified as a carrier, incidental, or secondary are listed in Supplementary Table 2.

**Table 1:** Chromosomal anomalies, diagnostic CNVs, and intragenic CNVs.

Patient No.	Pathogenic/likely pathogenic CNV	Size	Syndrome or critical region
79, 102, 240, 103, 350, 482	+18		Trisomy 18
155, 351, 438, 871, 747, 901	+13		Trisomy 13
827, 1008, 1142, 1506	+21		Trisomy 21
112, 772	45XO		Tuner syndrome
938	del(1p36.2p36.3).seq(823534-15632453)x1	14.8 Mb	1p36 deletion syndrome
864	dup(2q36.3q37.3).seq(226537458-242997727)x3	16.5 Mb	2q36.3q37.3 duplication
	del(6q26q27).seq(161507517-170879606)x1	9.4 Mb	Terminal 6q deletions
470	del(3q12.1q21.2).seq(99540858-125130561)x1	25.5 Mb	3q13.31 deletion syndrome region
244	del(4q31.3q32.1).seq(153436540-160087839)x1	6.6 Mb	
186	dup(5p14.3p15.3).seq(10429-23273021)x3	23.2 Mb	
	del(18p11.3p11.3).seq(111935-4272634)x1	4.2 Mb	
116	del(6q25.3q27).seq(160630268-170879606)x1	10.2 Mb	
456	dup(7p22.1).seq(5029498-6809995) x3	1.8 Mb	7p22.1 microduplication syndrome
190	del(7q35q36.3).seq(149299056-159068966)x1	9.8 Mb	Currarino syndrome
238	del(7q33q36.3).seq(137529688-159068966)x1	21.5 Mb	
422	del(7q35q36.1).seq(145049787-159068966)x1	14.0 Mb	
	dup(19q13.42q13.43).seq(55330867-59044235)x3	3.7 Mb	
653	del(8p11.21).seq(41835654-41836946)x1	1.3 Kb	KAT6A exon 6
532	dup(9p24.1p24.3).seq(10001-6476812)x3	6.5 Mb	
664	del(13q22.1q34).seq(74369596-115054392)x1	40.7 Mb	13q deletion syndrome
931	dup(13q31.2q34).seq(89863557-115054392)x3	25.2 Mb	Partial 13q trisomy
	del(20p13).seq(60001-2226344)x1	2.2 Mb	20p13 microdeletion syndrome
796	del(17p13.2p13.3).seq(1081133-4774754) x1	3.7 Mb	17p13.3 deletion syndrome
882	dup(17p13.3).seq(1150479-1592862) x3	442 Kb	17p13.3 microduplication syndrome
884	del(17q11.2).seq(29447084-29503935)x1	56.8Kb	NF1 microdeletion syndrome, NF1 exon 2-5
954	del(17q11.2).seq(228230706-228234866)x1‡	4.16 Kb	TM4SF20 exon 3
810	del(18p11.2p11.3).seq(111935-15323954)x1	15.2 Mb	
1004	del(18p11.2p11.3).seq(111935-15334797)x1	15.2 Mb

**Table 2:** Diagnostic SNVs identified by WGS.

No.	Gene	HGVSnom	Zygosity	Classification	Related disease and inheritance
49	KAT6B	c.3747delA(p. Gly1251Glufs*21)	Het	P	Genitopatellar syndrome, AD
432	STIL	c.3835 C > T(p. Arg1279Cys)	Het	LP	Microcephaly 7, primary, AR
	STIL	c.2344_2347delTTGC(p. Leu782Thrfs*2)	Het	P	
211	CC2D2A	c.3829 T > C(p. Cys1277Arg)	Het	LP	Joubert syndrome 9, AR
	CC2D2A	c.3874 G > T(p. Asp1292Tyr)	Het	LP	
234	KAT6B	c.3660dup(p. Arg1221*fs*1)	Het	P	Genitopatellar syndrome, AD
246	TUBA1A	c.748 G > T(p. Val250Phe)	Het	LP	Lissencephaly 3, AD
273	GJC2	c.1125_1135delCGGCCTCCCTG(p. Ala379Glyfs*109)	Het	LP	Lymphatic malformation 3, AD
348	DNM1L	c.345_346delAG(p. Glu116Lysfs*6)	Het	LP	Encephalopathy, AD
357	FGFR3	c.1948A > G(p. Lys650Glu)	Het	P	Thanatophoric dysplasia, AD
431	TUBA1A	c.614 A > T(p. Asp205Val)	Het	LP	Lissencephaly 3, AD
464	PDHA1	3:c.923_929delAGGAAGT(p. Ser312Valfs*1)	Het	LP	Pyruvate dehydrogenase E1α deficiency, XLD
479	GLI2	c.94dup(p. Ala32Glyfs*34)	Het	LP	Culler-Jones syndrome Holoprosencephaly 9, AD
558	PTPN11	c.1403 C > T(p. Thr468Met)	Het	P	Noonan syndrome 1, AD
627	NSD1	c.5177 C > T(p. Pro1726Leu)	Het	LP	Sotos syndrome, AD
648	FOXG1	c.171_180delCCCGCCGCCG(p. Pro60Argfs*129)	Het	LP	Rett syndrome, congenital variant, AD
730	FANCC	c.1330-1 G > A	Homo	LP	Fanconi anemia, complementation group C, AR
733	SIX3	c.339 G > A(p. Trp113Stop)	Het	P	Holoprosencephaly 2, AD
836	SOX2	c.480 C > G(p. Tyr160Stop)	Het	P	Optic nerve hypoplasia and abnormalities of CNS, AD
903	OFD1	:c.1103_1106delTGAT(p. Ile369Lysfs*18)	Het	LP	Orofaciodigital syndrome I, XLD
924	ARX	c.1074-1 G > A	Het	LP	Lissencephaly, XL2
942	NID1	c.1786C > T(P. Arg596*)	Het	LP	Dandy-Walker malformation, AD
993	PDHA1	c.933_936delAAGT(p. Ser312Argfs*13)	Het	P	Pyruvate dehydrogenase E1α deficiency, XLD
1278	CC2D2A	c.4333 C > T(p. Arg1445*)	Homo	P	Meckel syndrome 6, AR
222	PTCH1	c.2757_2758delCT(p. Phe919Leufs*39)	Het	LP	Basal cell nevus syndrome, AD
114	NF1	c.1742dup(p. Leu581Phefs*6)	Het	P	Neurofibromatosis, type I, AD

**Fig. 2:**
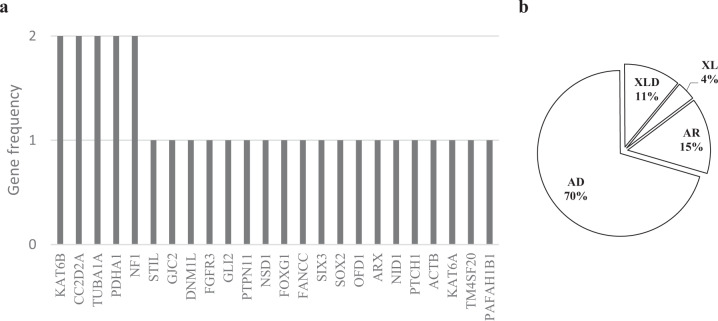
Critical genes identified in diagnosed cases and gene-related inheritance patterns. **a** Frequency of genes with diagnostic variants or critical genes in diagnostic CNVs. **b** Percentage of inheritance modes of diseases associated with genes in (**a**), AR autosomal recessive, AD autosomal dominant, XLD X-linked dominant.

The primary diagnostic yield of WGS varied significantly among the different ultrasound-defined CNS anomalies. As shown by the polyline in Fig. 2, hypoplasia of the cerebellum (including the cerebellar vermis) and holoprosencephaly had the highest primary diagnosis rates (>70%), followed by aplasia/hypoplasia of the corpus callosum, Dandy-Walker variants, intracranial cysts, and ventriculomegaly, with primary diagnosis yields of ~50%. Primary diagnosis yields for hydrocephalus, microcephaly, and undiagnosed abnormal echoes were between 20 and 30%. Unsurprisingly, primary diagnoses of destructive lesions and NTDs only reached ~10%. Diagnostic variants were present in 34 (70.8%) of the 48 fetuses with both CNS and non-CNS anomalies, and were present in 28 (24.6%) of the 114 fetuses with CNS anomalies only. There were 13 cases of isolated brain anomalies, including isolated ACC, cystic lesions, and ventriculomegaly, with six identified by primary genetic diagnosis. According to the detection limits of the variant size of common clinical genetic testing tools, such as karyotyping, CMA, and exome sequencing, the diagnostic variants were divided into three levels: i.e., chromosomal anomalies >5 Mb, CNVs between 100 kb–5 Mb, and single-gene variants, including SNVs and small CNVs. As indicated in Fig. 2, chromosomal anomalies and single-gene variants showed the greatest contribution to primary diagnosis in this cohort. In cases with only CNS anomalies, although the overall diagnosis rate was 24.6%, 15 (53.4%) out of 28 diagnosed cases were attributed to single-gene variants. Therefore, ES and deep WGS, which can detect single-gene variants, should be considered after uninformative clinical karyotyping for these types of anomalies.

As shown in Fig. 2a, among the 29 genes of diagnostic variants (26 SNVs and three established critical genes of diagnostic CNVs), five were found in more than one fetus (i.e., *TUBA1A*, *KAT6B*, *CC2D2A*, *PDHA1*, and *NF1*). Based on the OMIM database, 70% of these key 29 genes are related to autosomal dominant diseases and 15% are related to autosomal recessive diseases. The remaining 15% are X-linked or X-linked dominant, as shown in Fig. 2b. Protein-protein interaction analysis of these genes using the STRING database (http://string-db.org) showed a greater degree of interconnectivity than expected by chance (*P* = 1.75 × 10^−10^), and significant enrichment in brain development, forebrain development, and anatomical structure morphogenesis (Supplementary Fig. 3).

### Rare singleton deleterious variant enrichment

**Fig. 3:**
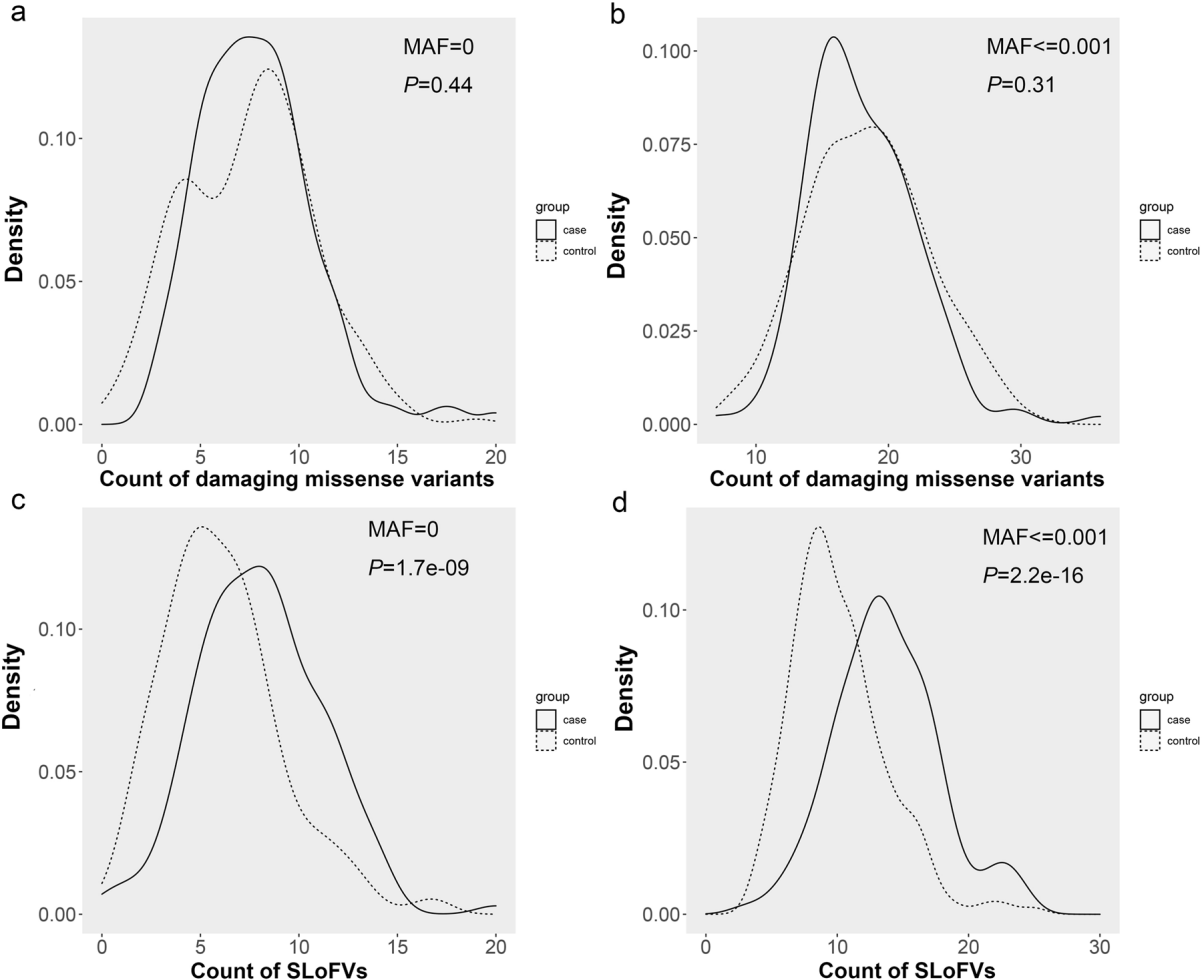
Accumulation of rare singleton deleterious variants in fetal CNS cohort. Distribution of rare singleton deleterious missense variants of **a** MAF = 0; **b** MAF ≤ 0.01. Accumulation of rare SLoFVs of **c** MAF = 0, with median of 8 in cases (solid line) and 6 in controls (dashed line) with *P* = 1.7 × 10^−9^ by two-sided Wilcoxon test and **d** MAF ≤ 0.01, with median of 13.5 in cases and 9 in controls with *P* = 2.2 × 10^−16^ by two-sided Wilcoxon test. SLoFVs singleton loss-of-function variants.

In total, SNVs in 136 cases were screened for singleton deleterious variants. Compared with the control group, singleton deleterious missense variants with different MAFs (0, 0.001) were not accumulated in the cohort (Fig. 3a, b). However, rare singleton loss-of-function variants (SLoFVs) with MAFs of 0 or <0.001 were significantly more common in the cohort than in the control group, as shown in Fig. 3c, d. The median number of rare SLoFVs (MAF ≤ 0.001) was 13.5 and 9 in the cases and controls, respectively, with *P* = 2.2 × 10^−16^ by the two-sided Wilcoxon test. These results suggest that the accumulation of rare SLoFVs may be related to fetal CNS anomalies and should be considered to improve diagnostic yield.

### Genomic architecture of NTDs

Despite being a common CNS abnormality both clinically and in this cohort, NTDs were found with a relatively low diagnosis rate, which is likely related to their complex etiologies, including various genetic and environmental factors^13,14^. Even so, pathogenic or likely pathogenic variants in four NTD cases were identified, including Trisomy 18, 7q36.1q36.3 deletion, 4q31.3q32.1 deletion, and homozygous nonsense c.4333C > T(p.Arg1445*) in CC2D2A causing Meckel Grubel syndrome in P1279.

The non-canonical Wnt/planar cell polarity (PCP) signaling pathway is implicated in the pathogenesis of NTDs, and 13 genes in the PCP pathway are involved in human NTDs^15^. Rare singleton deleterious variants in these 13 PCP genes (listed in Supplementary Table 3) were identified in two NTD cases, with none found in the control group. The two variants were NM_014246.1(CELSR1):c.7673G > A(p.Arg2558His) in P135 and NM_002336.2(LRP6):c.1252T > C(p.Tyr418His) in P382. In addition, known pathogenic missense mutation c.1744G > A(p.Gly582Ser) in COL3A1, which has been reported to cause Ehlers-Danlos syndrome IV^16^, was found in P175 with spina bifida occulta at the sacral vertebrae. In the second NTD case, P866 was diagnosed with exencephaly at 14 weeks, and a heterozygous missense variant with unknown significance in COL3A1 c.542 C > T(p.Pro181Leu) was also identified. Allele frequency of this variant in ExAC_EAS was 0.0002 and multiple lines of computational evidence supported a deleterious effect. Moreover, we found a canonical splice site mutation NM_000501.3:c.2032 + 1G > A in elastin (ELN) in P37 with microcephaly and meningocele. ELN is considered causative of autosomal dominant cutis laxa^17^ and supravalvular aortic stenosis^18^. COL3A1 and ELN are both related to vascular and skin pathologies and collagen and ELN are major structural components of the extracellular matrix^19^. Cutaneous vascular anomalies are associated with NTDs^20^. However, no further evidence is currently available to illustrate the relationships among variants in COL3A1 and ELN with NTDs.

### Imaging manifestations as phenotypes of molecular diagnosis

**Fig. 4:**
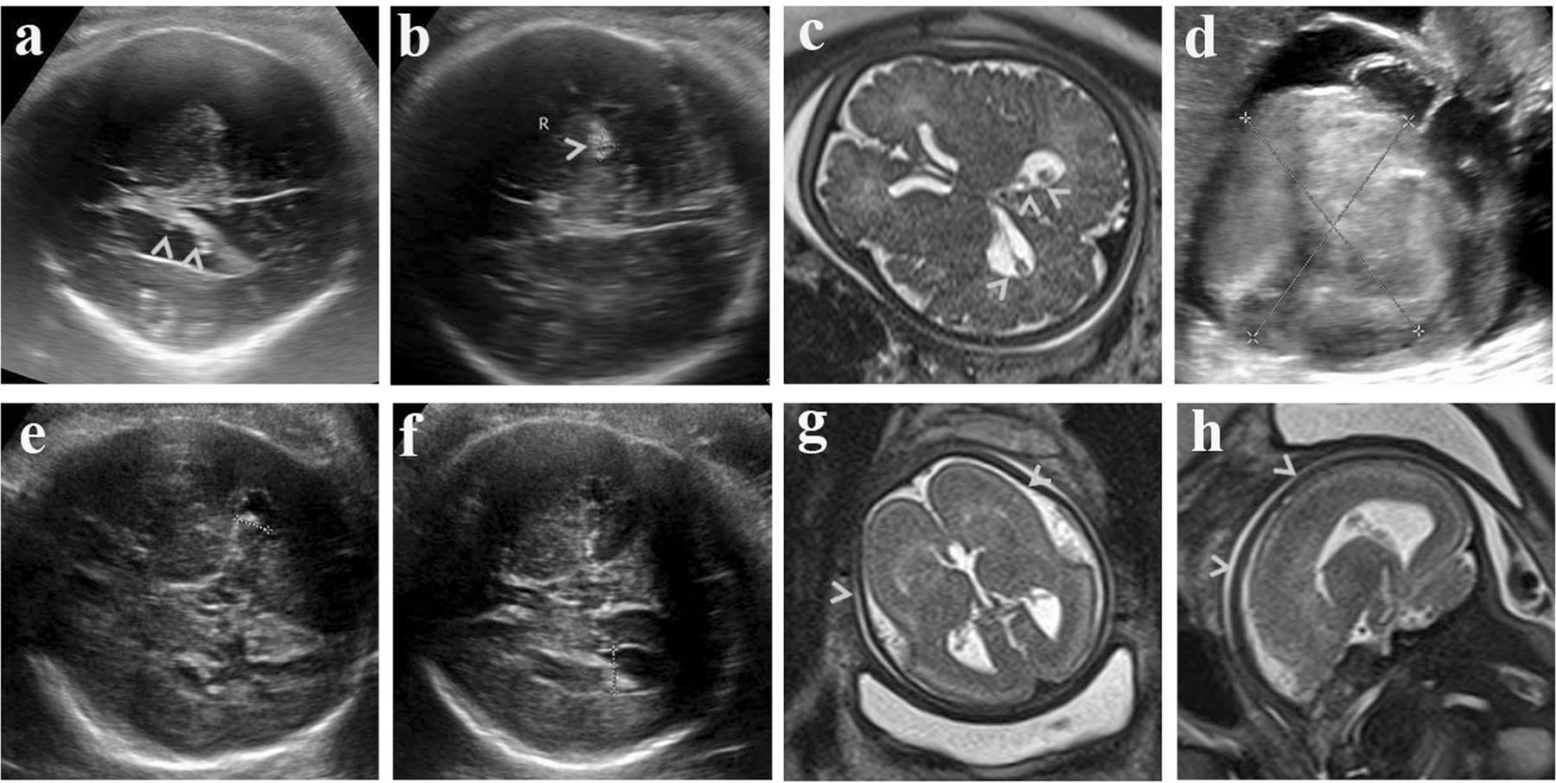
Prenatal imaging of two typical cases, P221 (top row) and P796 (bottom row). Sonographic hyperechoic lesions in **a** cerebral lateral ventricles and **b** brain parenchyma indicated by arrows. **c** Hypointensity lesions revealed by T2-weighted MRI in cerebral lateral ventricles indicated by arrows. **d** Large space-occupying lesion in left ventricle shown by **d** sonography. Widths of the cerebral ventricle of P796 were 0.84 cm on left (**e**) and 1.04 cm on right (**f**) shown by sonography. **g** Coronal and **h** sagittal view of T2 MRI showing abnormal cortical gyration indicated by arrows.

Patient phenotypes are extremely useful in narrowing candidate genes in untargeted sequencing such as ES and WGS^21^. Sequencing results are also of great significance for clarifying prenatal sonographic diagnosis. For example, P222 showed ventriculomegaly, hyperechoic lesions in the periventricular zone, and cardiac space-occupying lesions (suspected rhabdomyoma) accompanied by pericardial and pleural effusion, as seen in Fig. 4a–d. These findings were suspected to be tuberous sclerosis (TS) but ultrasound and MRI manifestation of the intracranial lesions were atypical. WGS identified no potential deleterious variants in TSC1 and TSC2. Instead, a frameshift mutation NM_000264.3:c.2757_2758delCT (p.Phe919Leufs*39) in PTCH1 was found, suggesting basal cell nevus syndrome rather than TS. Basal cell nevus syndrome is characterized by multiple nevoid basal cell epitheliomas and broad phenotypes, including cardiac fibroma and intracranial calcification^22^. In patient P954, a complex-4 160-bp deletion (2: 228230706-228234866) in the *TM4SF20* gene was found, which consisted of two microdeletions separated by a 100-bp gap. Prenatal sonography of the fetus showed a widened left lateral ventricle and abnormal hypoechoic signals around the bilateral anterior horns. This intragenic ancestral deletion is reported to be segregated with early language delay disorders and cerebral white matter hyperintensity (WMH) in 15 unrelated families predominantly from Southeast Asian populations^23^. In that study, one severely affected patient, born prematurely at 33-weeks of gestation, presented gross enlargement of the third and lateral ventricles with near-complete loss of periventricular white matter, while other patients showed milder but varying degrees of WMH. In the present study, variants of NF1 were found in two cases, i.e., P114 and P884. Ultrasonography revealed that P114 had hydrocephalus and clubfoot, whereas P884 was found to have enlarged lateral and third ventricles, a missing septum pellucidum, and a non-echoic lesion at the cerebral midline, and was therefore diagnosed with corpus callosum hypoplasia and midline cyst. Previous studies have found ventriculomegaly, generalized enlargement of the pericerebral spaces, and dilated ventricles in fetuses, as well as Chiari I with hydrocephalus, septum cavum pellucidum, and agenesis of the corpus callosum in patients with NF1 microdeletions and variants^24–26^. Skeletal abnormalities such as osteopenia/osteoporosis, shortness of stature, scoliosis, and congenital pseudarthrosis of the tibia are frequently associated with neurofibromatosis 1^27^. Mensink et al.^25^ reported unilateral clubfoot in a patient with a 1.94-Mb deletion covering the entire NF1 gene. Therefore, although not specific, prenatal findings may be associated with common NF1 phenotypes.

### Cost and turnaround time with WGS

Chung et al.^28^ reported that the cost of prenatal CMA in Hong Kong is US$628. Li et al.^29^ reported that the cost of CMA in patients with unexplained global developmental delay or intellectual disability in the USA is US$646. Tsiplova et al.^30^ stated that the cost of CMA for autism spectrum disorder is US$580. Therefore, the costs of CMA in different areas for various applications are relatively similar. At the same time, as shown by Schwarz^31^, the costs for a single WES or WGS analysis range from US$555 to US$5,169 and from US$1906 to US$24,810, respectively. Previous comparisons of the single-test costs of WES and WGS found that WGS (HiSeq X platform) is more expensive than WES (US$933 and US$921 in the two studies, respectively), though the average costs for both were different^30,32^. The main differences come from the capital costs of sequencing platforms, contract maintenance with platform suppliers, sequencing consumables and reagents, and bioinformatics. These are also the main costs involved in genome sequencing. At present, the costs of using the HiSeq X Ten^33,34^ and BGISEQ500 platforms for human WGS are US$1000 and US$600, respectively.

Using the pipeline described in the methods of this study, our turnaround time was approximately three weeks. Two days were required for DNA extraction and sequencing library construction. Five to seven days were required for genome sequencing to produce 40X raw data on the BGISEQ500 platform. Bioinformatics analysis, including alignment, variant calling, and annotation, required one to two days, and manual interpretation and reporting required one day. Sanger validation required one week as it was outsourced.

Currently, the turnaround time of prenatal CMA is ~15 working days, although it can be shorter or longer depending on operational protocols in different labs. The turnaround time of clinical proband WES is around 12–15 weeks^35,36^, but the turnaround time can be reduced to 2–4 weeks using trio WES or rapid bioinformatics pipelines in situations requiring fast assessment, such as prenatal, neonatal, or pediatric intensive care diagnosis^35,37^. Indeed, 24 h trio-ES or 26 h WGS pipelines are available for critically ill infants^38^.

## Discussion

In this cohort of pregnancies with fetal CNS anomalies, a primary genetic diagnosis was achieved in 38.3% (62/162) of cases based on WGS. Although commonly used CMA plus ES was not applied here for direct comparison with WGS, several previous studies on diagnostic yields of genetic testing at different resolution levels were reviewed and those with relatively large sample sizes are summarized in Table 3^7,8,39–42^. At the microscopic level, we found that the low-pass WGS diagnostic rate was close to that of karyotyping, although balanced translocation and triploid analyses were not applicable using our WGS approach. Among the cases without microscopic genetic diagnosis, low-pass WGS found additional causative CNVs in 2.3% of cases at the sub-microscopic level. This is lower than that reported by Shaffer et al.^41^, which could be due to the higher proportions of different CNS ultrasound phenotypes found in our study compared to other research. In our cohort, NTDs and hydrocephalus were the two most common phenotypes, while posterior fossa defect was the least common; in contrast, NTD and hydrocephalus had the lowest diagnosis rate, while posterior fossa defect had the highest diagnosis rate, consistent with Shaffer et al.^41^. In addition, in Shaffer’s study, the resolution of conventional karyotyping was set to 10 Mb, while we set the extra diagnosis rate to 5 Mb.

**Table 3:** Comparison of diagnostic yields for variants at different resolutions.

Variant resolution	Diagnostic yield (primary diagnosis/total cases)							
	Publications					Present study		
	CNS only	CNS + other		CNS total		CNS only	CNS + other	CNS total
*Microscopic level*								
Chromosomal anomaly	8.5% (77/908)^40^		NA		18.4% (37/201)^42^	9.6% (11/114)	43.8% (21/48)	19.8% (32/162)
Balanced translocation, triploid	0.4% (4/908)^40^		NA		2% (4/201)^42^	NA	NA	NA
Submicroscopic level	5.0% (19/382)^41^		6.0% (19/317)^41^		5.4% (38/699)^41^	1.9% (2/103)	3.7% (1/27)	2.3% (3/130)
SNV	23.1% (15/65)^40^		NA		22% (11/49)^7^ 4.3% (3/69)^8^	11.9% (12/101)	46.2% (12/26)	18.9% (24/127)
Intragenic CNV (50 bp–100 kb)	NA					3.4% (3/89)	0	2.9% (3/103)

*NA* not applicable.

As shown in Table 3, diagnostic SNVs were found in 14.8% of the cohort, which increased to an 18.9% diagnostic rate after uninformative low-pass WGS. However, ES diagnostic yields of fetal anomalies show considerable variation. For example, in a prospective cohort study of 243 maternal-fetal samples, Petrovski^7^ reported an over-diagnosis rate of 10% among fetuses with prenatal structural abnormalities based on trio WES, with diagnostic genetic variation in the CNS subgroup of 22% (11/49). In another large prenatal ES study of 610 nuclear families, Lord et al.^8^ reported a diagnostic yield of 4.3% in 69 fetuses with brain anomalies. Fetuses with abnormal karyotypes or relevant CNVs were excluded in both studies. These variations among studies may be due to different sample sizes, fetal phenotype compositions, or criteria of variant classification and patient exclusion. In the current study, the fetuses were studied retrospectively, rather than selected based on previous genetic results or genetic etiology. This was because the main purpose of our research was not only to evaluate the diagnostic capabilities of WGS, but also to demonstrate the genomic architecture of common abnormal ultrasound manifestations, and thus ensure that appropriate genetic testing tools are applied in prenatal settings and screenings.

It is worth noting that intragenic CNVs and their possible causal relationships with fetal anomalies were systematically evaluated in the current study. Although several genetic testing panels designed for specific diseases may involve probes or primers for detecting intragenic CNVs, they have not been used in a wide range of disease-related genes. Their application scope is even more limited prenatally as fetal abnormalities are difficult to diagnose differentially and the genetic background of fetal structural anomalies is highly heterogeneous. Although WES has become a primary strategy for sequencing in patients with suspected genetic diseases, CNV detection remains challenging due to its technological characteristics. Compared with CNVs identified using deep WGS data from the same sample, common algorithms of CNVs calling using WES data suffer from limited power^43^. However, intragenic CNVs may account for a substantial proportion of pathogenic variants. Truty et al.^44^ investigated the prevalence and properties of intragenic CNVs in >143,000 individuals referred for genetic testing and found a 10% prevalence of intragenic CNVs among individuals with a positive test result and high frequencies in neurological diseases. In this study, intragenic CNV analysis identified three possible pathogenic intragenic CNVs, resulting in an additional diagnosis rate of 2.9%. In addition, we identified other endogenous CNVs encompassing internal exons and predicted that they would adversely affect the transcriptional reading frame of genes with loss-of-function mechanisms. However, we found no conclusive evidence that they were related to abnormalities in the fetal CNS. Therefore, additional WGS studies are required to establish prenatal ultrasound manifestations with intragenic CNVs.

The HPO database can be used to select candidate genes^10^. However, prenatal ultrasound results of the brain may be non-specific or evolving, and minor abnormalities may be missed or undiagnosed. For example, although experienced experts can detect signs of abnormal cortical development through advanced neurosonography and MRI, these anomalies are difficult to confirm mid-trimester. In the current study, using WGS, enlargement of the lateral ventricle and subarachnoid space was confirmed to be lissencephaly (e.g., in P431 and P796). Therefore, to enhance genetic diagnosis of abnormalities in the fetal CNS, describing abnormal ultrasound findings may be more useful than performing disease diagnosis.

An increased burden of rare deleterious variants in genes, pathways, and genomes has been found in neurological and developmental disorders, such as schizophrenia^45^, epilepsy^46^, and autism spectrum disorder^47^. In this study, rare singleton deleterious variants, including predicted missense and loss-of-function variants with different MAF frequencies (i.e., <0.01, 0.001, 0), were examined in the 136 cases and 200 controls. Compared with the control group, only rare SLoFVs were statistically enriched in the cohort. Chen et al.^48^ has reported the accumulation of rare SLoFVs in NTDs. In our study, despite this trend, the increase in SLoFVs in NTDs was not significant, which may be due to the small NTD sample size in our study.

Though WGS has an absolute advantage in variant detection, there has long been concern that WGS may require more fetal DNA, longer turnaround time, and higher cost^8^. These concerns were not warranted. The required DNA depends on the library construction protocols, not sequencing itself. Usually, 1 μg of genomic DNA is used as the standard protocol for testing multiple products across popular platforms^49^, with some kits requiring even less DNA. The TruSeq Exome Library Prep protocol (November 2015) uses 100 ng of starting DNA with Covaris fragmentation. The MGI Easy Universal DNA Library Prep Set requires only 0.5–50 ng if fetal DNA is extremely limited. Furthermore, WGS does not need additional DNA for CNV analysis, because both low-pass and deep WGS only require a single WGS sequencing library. In regard to turnaround time, it usually takes 2–15 weeks to obtain and interpret ES results^7^. In contrast, the WGS turnaround time in our study was 2–3 weeks per sample for interpretation of chromosomal anomalies, CNVs, SNVs, and small CNVs, and another week for Sanger validation. Thus, for routine protocols, WGS is as fast as ES, and if the extra time required for CMA is considered as a prerequisite for ES, WGS is even faster. In addition, on the BGI platform, the sequencing costs of WGS are lower than those of CMA plus ES, given that CMA costs about US$600. In view of the decline in the cost of human genome sequencing, the price difference between WGS and WES is now about US$600. For example, Illumina introduced US$600 genome sequencing with the launch of its NovaSeq^TM^ 6000 v1.5 kit in 2020^50^. BGI currently offers US$500 genome sequencing with its T7 platform and has introduced US$100 genome sequencing with its new DNBSEQ Tx platform and CoolMPS sequencing kits^51^.

Although WGS is a suitable method for identifying fetal CNS anomalies, our approach has some limitations. Firstly, triploids were not analyzed in this study. Furthermore, due to the lack of large-scale databases, it was difficult to determine the clinical significance of variants in introns and noncoding areas. Even though we did find some variants in introns that were previously reported as likely pathogenic, we considered that they were more likely to be benign due to their high frequency in the control database. As a continuation of this preliminary study on abnormalities in the CNS, we are now studying more cases of fetal ultrasound abnormalities in each anatomical system, which should hopefully further prove the potential power of WGS in genetic screening.

## Methods

### Patient identification, ultrasound examination, and sample collection

Patients were found by a multi-level referral system of prenatal screening and diagnosis between 2015 and 2017. This referral system was launched by the Maternal and Child Health Hospital of Hubei Province (MCHH, China) and includes 156 prenatal screening units and 32 diagnostic centers across Hubei and several districts in middle and southwestern China. All screening units and diagnostic centers received training from the MCHH following the practice guidelines of the International Society of Ultrasound in Obstetrics & Gynecology (ISUOG)^52^. If fetal structural anomalies were detected during routine ultrasound scans at any of these units or centers, patients were transferred to the MCHH for systematic prenatal ultrasound by at least two chief physicians to obtain a final diagnosis. MRI was further provided upon patient/family acceptance. Those patients who donated invasive testing samples, abortion samples, or birth samples were invited to participate in a congenital defect study. Here, patients diagnosed with fetal CNS anomalies were identified from the congenital defect study by reviewing medical records and images. In total, 162 patients who provided a fetal sample and written informed consent were included (161 with umbilical cord tissue samples collected after termination of pregnancy, and one amniotic fluid sample). Demographic information was asked of the pregnant women, but they could refuse to answer. WGS was performed after pregnancy outcome (i.e., abortion or live birth), and thus outcome was not influenced by the WGS results. This project followed Chinese state and local regulations related to biological and medical research and was approved by the MCHH and BGI Ethics Committee (BGI-IRB17039).

### Whole-genome sequencing

Genomic DNA from fetal tissue was extracted using the salting-out method, as described in Miller et al.^53^. To be simplified, approximately of 200 mg tissue was diced and homogenated with 2 ml of lysis buffer(10 mM Tris, 400 mM NaCl and 2 mM Na_2_EDTA). Then the tissue lysates were incubated at 65°C with 0.2 ml of 10% SDS and 0.5 ml of a protease K solution (1 mg protease K in 1% SDS and 2 mM Na_2_EDTA) for 30 min. After incubation, 2 ml of 6 M NaCl was added and vigorously shaken several times, followed by centrifugation at 12,000 rpm for 5 min. The supernatant was transferred and 2 volumes of absolute ethanol was added. The precipitated DNA strands were dissolved in 100–200 μL TE buffer and examined using a Qubit 3.0 fluorometer (Life Technologies, Paisley, UK) and 1% agarose gel electrophoresis. A sequencing library was constructed and sequenced using the BGISEQ500 or MGISEQ2000 sequencer platforms (MGI, Shenzhen, China) according to the manufacturers’ instructions^54^. The paired-end read length was 100 bp. All 162 samples were first subjected to low-pass WGS (~0.5X). If aneuploids or diagnostic CNVs (>100 kb) were not identified, the sequencing library was additionally sequenced to a 40X depth for identification of single-nucleotide variants (SNVs, including insertions/deletions (indels)) and small CNVs (50 bp–100 kb).

### Variant calling and annotation

Chromosomal anomalies, including aneuploids and rare CNVs, were called based on the pipeline developed by Dong et al.^55^. Deep sequencing data were aligned to the human reference genome GRCh37/hg19 and SNVs were called and filtered using the Edico Genome DRAGEN Bio-IT Platform^56^. Small CNVs were called using the SpeedSeq SV pipeline (v.0.0.3a)^57^. Small CNVs and SNVs were annotated using bcfanno (v1.4) (https://github.com/shiquan/bcfanno) with frequencies from several public databases (ExAC^58^, GnomAD^59^, and G1000^60^) and an in-house population database containing 790 samples sequenced to ~40X depth using the same sequencing and analysis platforms.

### Primary diagnosis

Variants were interpreted by a clinical review panel consisting of geneticists, senior sonographers, MRI radiologists, and obstetrician gynecologists to decide the clinical significance and relevance of the fetal imaging phenotypes.

First, aneuploids and CNVs were manually classified by geneticists into five categories: i.e., pathogenic, likely pathogenic, uncertain significance (VUS), likely benign, and benign, as per the American College of Medical Genetics and Genomics (ACMG) guidelines for the evaluation of CNV pathogenicity: pathogenic ≥0.99, likely pathogenic 0.90–0.98^61^. Pathogenic or likely pathogenic CNVs were further evaluated if they showed evidence related to fetal imaging manifestations. Relevant pathogenic or likely pathogenic CNVs were designated as primary diagnostic variants.

The SNVs were filtered before manual interpretation. First, SNVs with minor allele frequencies (MAF) >0.01 in the public and in-house databases or in introns and noncoding areas were filtered out. Imaging manifestations for each fetal sample were transformed to related Human Phenotype Ontology (HPO) terms^62^. Genes related to HPO terms in the HPO database formed a fetus-specific candidate gene list. SNVs not found on any gene or not on genes in the candidate gene list were filtered out. The remaining SNVs underwent the same diagnostic assessment as CNVs but the classification criteria followed the ACMG guidelines for SNV interpretation^63^.

If primary diagnostic SNVs were not identified, then the interpretation processes of CNVs were applied to small CNVs to determine primary diagnosis.

### Incidental and secondary findings

Variants previously classified as pathogenic/likely pathogenic in the ClinVar Archive (https://www.ncbi.nlm.nih.gov/clinvar/) or as protein-truncating variants affecting a known disease gene via a loss-of-function mechanism but lacking evidence to support its association with prenatal phenotypes were classified as incidental findings. Secondary diagnostic findings were defined as incidental findings based on the ACMG recommendation list^64^.

### Rare singleton deleterious variant analysis

The SNVs identified by deep WGS were filtered to retain high-quality variants only. The filtering criteria were read depth ≥ 20, altered allelic read depth ≥ 3, and variants ≤ 5 bp. Deleterious variants included missense variants predicted to be damaged based on the following prediction algorithms: SIFT^65^ and CADD score > 20^66^, M-CAP > 0.025^67^, and REVEL > 0.5^68^, as well as loss-of-function variants, including nonsense, frameshift, canonical splice sites, and stop gain/loss. Singleton variants were those identified only once in the cohort. The number of singleton deleterious variants with MAFs from 0 to 0.001 in the public databases were calculated and compared in cases and controls. The case group consisted of 136 samples in the cohort where deep WGS data were available, and the control group consisted of 200 randomly selected individuals from the in-house population database mentioned above.

### Validation

Primary pathogenic or likely pathogenic SNVs were validated by Sanger sequencing. Microduplications were validated by quantitative polymerase chain reaction (qPCR) and microdeletions were validated by PCR and agarose gel electrophoresis. Primers were listed in Supplementary Table 6.

### Reporting summary

Further information on research design is available in the Nature Research Reporting Summary linked to this article.

## Supplementary information

Supplementary materials

Reporting Summary Checklist

## Data Availability

Data are uploaded to NCBI with accession number PRJNA798107. Variation data can be accessed in dbSNP with the link https://www.ncbi.nlm.nih.gov/SNP/snp_viewTable.cgi?handle=YY_MCH. Data are also publicly available in the China National GeneBank Database (CNGB) Nucleotide Sequence Archive (Accession Number: CNP0002431).

## Code availability

All analyses in this study were performed using Edico Genome v2.4.1, SpeedSeq SV pipeline (v.0.0.3a). bcfanno (v1.4), R version 3.5.2, Python 3.6. Custom R and Python scripts used for the singleton variant analyses are available upon request.
