# Supplementary material for: Genomic architecture of fetal central nervous system anomalies using whole-genome sequencing
Source: NPJ Genom Med. 2022 May 13;7:31. doi: 10.1038/s41525-022-00301-4 (PMC9106651; doi:10.1038/s41525-022-00301-4)
Supplement: Supplementary file 1 — Supplementary materials [file 41525_2022_301_MOESM1_ESM.pdf]

Supplementary Materials

Supplementary Figure 1 Typical types of fetal CNS anomalies and case numbers in the cohort 1
Supplementary Figure 2 Distribution of small CNVs identified by deep WGS 2
Supplementary Figure 3 Protein-protein interaction and enrichment of causative genes 3
Supplementary Table 1 Clinical information of patients 4
Supplementary Table 2 Candidate variants and Incident findings 15
Supplementary Table 3 Planar cell polarity gene list 16
Supplementary Table 4 CNVs pathogenicity interpretation criteria 17
Supplementary Table 5 SNVs pathogenicity interpretation criteria 19
Supplementary Table 6 Primer List 23

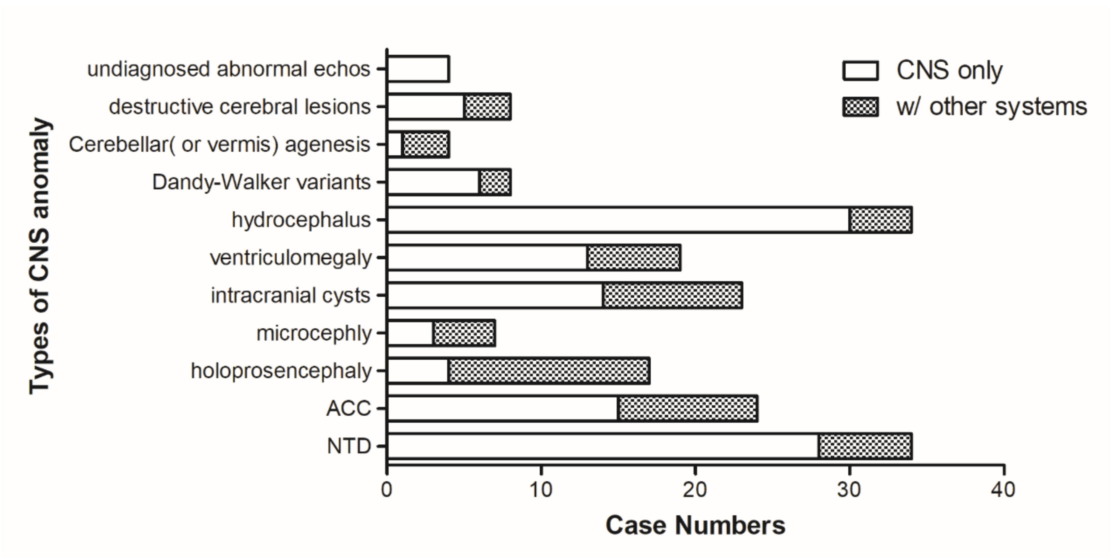

Supplementary Figure 1 Typical types of fetal CNS anomalies and case numbers in the cohort

Blank bars indicated fetuses having CNS malformations only (confirmed by MRI or autopsy). Dotted bar indicated cases with additional anomalies outside of CNS. Cases counted more than once if multiple types of CNS anomalies occurred concurrently. ACC: aplasia of corpus callosum; NTD: neural tube defects.

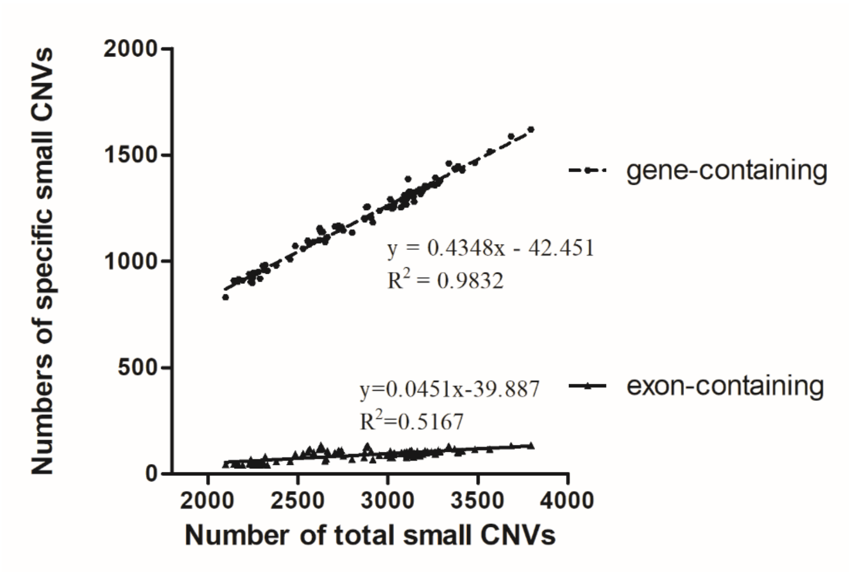

Supplementary Figure 2 Distribution of small CNVs identified by deep WGS

Numbers of total small CNVs are linearly correlated with those of gene-containing and exon-containing small CNVs.

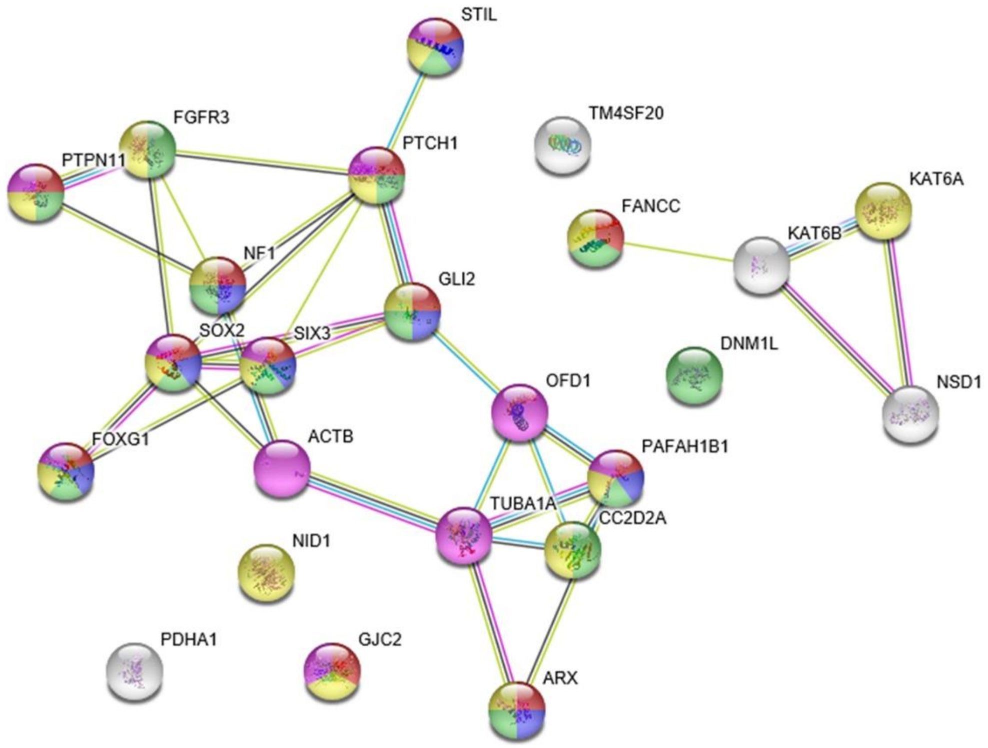

Biological Process (GO)			
GO-term	description	count in gene set	false discovery rate
GO:0007420	brain development	12 of 650	6.03e-09
GO:0030900	forebrain development	8 of 366	3.71e-06
GO:0009653	anatomical structure morphogenesis	14 of 1992	3.71e-06
GO:0048513	animal organ development	16 of 2926	3.80e-06
GO:0051726	regulation of cell cycle	11 of 1129	7.02e-06

Supplementary Figure 3 Protein-protein interaction and enrichment of causative genes

Colors of the nodes showing the biological process the genes fit in. The top-5 enrichment biological process was brain development, forebrain development, anatomical structure morphogenesis, animal organ development and regulation of cell cycles. The analysis was done by STRING database (http://string-db.org) with a significant p-value of 1.75×10^{-10}.

Supplementary Table 1 Clinical information of patients

Patient No.	Age	Pregnancy History	Sonographic diagnosis	Clinical gestation	Ultrasound estimated gestation	Sex	Malformation at birth or TOP*	Additional finding by MRI or autopsy
41	26	G1P0	echogeneous foci at sacral vertebrae	28.4	27.6	F	spina bifida occulta	No
42	24	G3P0	subependymal cysts, arachnoid cysts	38.4	38	M	NA	No
45	24	NA	Chiari II malformation	23.1	23.2	M	Scoliosis, open spina bifida, brain autopsy unavailable	No
47	18	NA	Chiari II malformation	21.3	21.2	F	lemon-shaped head, open spina bifida, rocker bottom foot	No
49	26	G1P0	hypoplasia of corpus callosum, renal pelvis dilation, bilateral clubfeet	24.3	23.6	M	NA	No
53	21	G1P0	anencephaly, spina bifida	38.2	34.1	M	anencephaly, spina bifida	No
54	32	NA	hydrocephalus	21.5	21.6	M	NA	No
62	27	NA	hydrocephalus	27.2	27.1	M	NA	No
63	33	NA	unilateral hydrocephalus, arachnoid cysts	30.6	32.1	M	NA	No
69	27	G1P0	aplasia of corpus callosum	24.5	24.4	M	NA	No
79	31	NA	low-set ear, choroid plexus cysts, Chiari II malformation, ventricular septal defect, single umbilical artery, clubfeet	22.5	20.2	F	open spina bifida, clubfoot, autopsy of heart unavailable	No
80	26	NA	hydrocephalus	22.6	22.1	F	NA	No
83	24	G1P0	anencephaly	38.3	34.6	M	anencephaly	No
85	31	G2P0	hydrocephalus	38.4	37.5	F	NA	No

86	29	G1P0	choroid plexus cysts, bilateral renal agenesis	23	21.2	M	NA	No
96	23	NA	aplasia of corpus callosum, median arachnoid cysts	27.5	27.5	M	aplasia of corpus callosum, periventricular heterotopia (MRI)	Yes
97	26	NA	hydrocephalus	20.6	19.6	F	NA	No
101	30	NA	hydrocephalus, calcification, intracranial hemorrhage	33.2	32.4	F	NA	No
102	29	G4P0	lemon-shaped head, choroid plexus cysts, nuchal lymphatic cysts, VSD, coarctation of the aortic arch, persistent left superior vena cava	16.6	16.4	F	lemon-shaped head, nuchal lymphatic cysts, VSD	No
103	28	No	choroid plexus cysts, low-set ear, VSD	25.4	24.3	M	NA	No
112	25	G1P0	aplasia of corpus callosum	24.3	25.3	F	NA	No
114	32	G3P1	hydrocephalus, clubfeet	20.5	12.1	M	hydrocephalus, clubfoot	No
116	23	NA	hydrocephalus	37.4	37.5	F	NA	No
121	27	G1P0	intracranial hemorrhage	33	33.6	M	intracranial hemorrhage	No
135	28	G2P0	exencephaly	35.1	33.5	F	NA	No
136	27	G2P0	ventriculomegaly	32.1	33.5	F	NA	No
138	28	G1P0	microcephaly, meningoencephalocele	17.3	16.3	F	microcephaly, meningoencephalocele, finger and toe syndactyly	Yes
141	22	G1P0	Chiari II malformation	25.4	23.2	F	spina bifida, tethered spinal cord	No
149	40	G2P1	hydrocephalus, hypoechoic lesions (suspected arachnoid cysts)	27.2	29	M	hydrocephalus, brain edema	No

155	26	G2P0	Dandy-Walker malformation, aplasia of corpus callosum, oral cleft, polydactyly, rocker-bottom foot, esophageal atresia	24	23.6	M	NA	No
156	33	G2P1	aplasia of corpus callosum, arachnoid cysts	27.1	26.3	F	aplasia of corpus callosum, arachnoid cysts, parietal frontal dysplasia (MRI)	Yes
160	29	NA	ventriculomegaly, micrognathia, low-set ears, clubfeet	27	27.5	M	absent of tongue, absent nares, low-set ears	No
164	37	G2P0	ventriculomegaly	25.5	26.2	F	NA	No
169	26	G2P0	hypoplasia of corpus callosum	37.1	37	M	NA	No
170	26	NA	hydrocephalus	34.5	35.6	F	NA	No
173	24	G1P0	Microcephaly, micrognathia, abnormal limb posturing, scoliosis	36.1	27.5	F	microcephaly, micrognathia, limb rigid, hip dislocation, sacrococcygeal hypoplasia	No
175	24	G1P0	spina bifida	27.3	27.1	M	spina bifida	No
186	24	G2P0	holoprosencephaly， cyclopia, proboscis	25.3	20.2	F	holoprosencephaly, cyclopia, proboscis	No
190	32	NA	spina bifida occulta	31.3	30.5	F	spina bifida occulta	No
197	21	G1P0	microcephaly	35.4	31.1	F	microcephaly	No
211	27	G2P0	absence of cerebellar vermis	23.5	24.2	M	absence of cerebellar vermis	No
219	28	G1P0	arachnoid cysts	36.6	36.6	F	NA	No
222	21	G1P0	Ventriculomegaly, periventricular hyperechoic lesions, cardiac space-occupying lesions	40.4	40.4	F	intracranial calcification lesions, cardiac fibroma	No
227	31	G1P0	Dandy-Walker malformation	23.4	20.5	M	NA	No

231	32	G5P1	ventriculomegaly, IUGR	37.2	32.2	M	NA	No
233	18	G2P0	intracranial hemorrhage	37	37.5	M	intracranial hemorrhage	No
234	35	G2P1	aplasia of corpus callosum, renal pelvis dilation, clubfeet	23.3	22.1	M	aplasia of corpus callosum, absent septum pellucidum, clubfeet	No
236	25	G3P0	Chiari II malformation	26.6	27.2	M	NA	No
238	25	G1P0	holoprosencephaly, facial anomalies, heart defects	18.3	17.4	F	NA	No
240	27	G2P1	holoprosencephaly, facial anomalies, abnormal hands and feet posture	35.4	31.3	F	NA	No
241	24	G1P0	hypoplasia of corpus callosum, hydrocephalus	35.6	35.2	F	NA	No
243	29	G4P1	aplasia of corpus callosum, arachnoid cysts	26.3	26.3	M	NA	No
244	29	G1P0	meningocele	15.6	14.1	M	NA	No
246	23	G1P0	microcephaly, arachnoid cysts, micrognathia	28.2	24.4	F	NA	No
257	28	G1P0	cystic lesions above cerebellar tentorium	38	38.3	M	NA	No
258	25	G2P1	hydrocephalus	25.2	25.2	M	hydrocephalus	No
262	22	G1P0	intracranial hemorrhage	35.5	37	F	NA	No
268	27	G1P0	hydrocephalus	33.4	33.1	M	NA	No
269	31	G2P0	subependymal cysts	36.2	35.2	M	NA	No
270	22	G1P0	anencephaly, body stalk anomaly	15.6	14.4	F	anencephaly, body stalk anomaly	No
273	30	G1P0	cystic lesions above cerebellar tentorium	32.2	33.2	F	NA	No

274	29	G1P0	anencephaly	23.1	22.5	F	anencephaly	No
280	26	G1P0	Dandy-Walker malformation	22.1	22.3	F	Dandy-Walker malformation	No
284	31	G1P0	Dandy-Walker malformation	22	21.3	F	NA	No
286	28	NA	ventriculomegaly, cystic lesions at posterior horns of the right lateral ventricle	35.4	35.4	F	NA	No
296	22	G1P0	hydrocephalus, hypoechoic lesions in and around left lateral ventricles (suspected intracranial hemorrhage)	35.1	33.3	F	NA	No
330	27	G1P0	aplasia of corpus callosum, periventricular hyperechoic lesions	33	34.5	F	NA	No
335	21	NA	hydrocephalus, subependymal cysts	32.3	32.1	F	NA	No
341	39	NA	hydrocephalus	34	35.5	M	NA	No
342	25	G1P0	holoprosencephaly	22	21.4	F	NA	No
346	25	G2P0	hydrocephalus	25.6	26.1	F	NA	No
348	23	NA	ventriculomegaly, congenital cystic adenomatoid malformation of the lung	28.5	29.6	M	NA	No
350	24	G2P0	hypoplasia of corpus callosum, endocardial cushion defect	27.2	25.5	M	NA	No
351	24	G2P1	Dandy-Walker malformation, increased renal parenchymal echogenicity	26.1	26.3	M	NA	No
353	26	G1P0	hydrocephalus	36.4	37	M	NA	No

357	30	G2P0	hypoplasia of corpus callosum, cerebellum hypoplasia, short limbs	24	22.1	M	NA	No
382	27	G3P0	anencephaly	21.3	21.3	F	NA	No
407	23	G1P0	myelomeningocele, clubfeet	23.5	21.6	M	NA	No
413	28	G1P0	hydrocephalus, subependymal cysts	36.1	37.1	M	NA	No
422	29	G2P0	Dandy-Walker malformation	21	19	M	NA	No
431	26	G1P0	ventriculomegaly	33.2	29.6	M	NA	No
432	33	G5P0	microcephaly	22.5	20.4	M	NA	No
438	27	G2P1	nuchal lymphatic cysts, holoprosencephaly, diaphragmatic hernia	14.2	12.6	M	NA	No
456	28	G2P1	nuchal lymphatic cysts, ventriculomegaly, choroid plexus cysts, diaphragmatic hernia, overlapping fingers, single umbilical artery	25.1	24.4	M	NA	No
464	29	G3P1	hydrocephalus	33.4	29.6	F	NA	No
470	33	G5P0	aplasia of corpus callosum	36.3	34.3	F	NA	No
471	24	G1P0	hydrocephalus	36.1	37.5	M	NA	No
473	25	G2P0	intracranial hemorrhage	31	31	M	NA	No
479	28	NA	arachnoid cysts	24.5	24.2	M	6*3cm arachnoid cysts beside right lateral ventricle	No
481	28	G1P0	spina bifida occulta	25.4	25.6	M	NA	No
482	24	G1P0	aplasia of corpus callosum	23.3	23.2	M	NA	No
493	26	G2P0	hydrocephalus, oligohydramnios	23	22.2	F	grey spots lesion on liver surface, brain autopsy unavailable	No

494	28	G3P1	subependymal cysts	36	34.1	M	brain autopsy unavailable	No
497	27	NA	Chiari II malformation	23	21.4	F	Chiari II malformation	No
532	24	NA	hydrocephalus, subependymal cysts	33	35	F	NA	No
542	19	NA	spina bifida, diastematomyelia	36.4	35	F	diastematomyelia	No
548	35	G1P0	exencephaly	15	15.2	M	exencephaly	No
558	26	G4P2	hydrocephalus	34.3	34.4	F	NA	No
563	28	NA	hydrocephalus	35.1	32.3	M	NA	No
572	23	G2P1	hydrocephalus, hypoechoic lesions in lateral ventricles (suspected intracranial hemorrhage)	38.4	38.3	M	pericardial effusion, brain autopsy unavailable	No
578	24	G1P0	hypoplasia of corpus callosum, holoprosencephaly	26.1	25.1	M	syndactyly, oligodactyly, short fingers, brain autopsy unavailable	Yes
602	25	G1P0	exencephaly	13.6	13.5	F	exencephaly	No
627	33	NA	nuchal lymphatic cysts, subependymal cysts	37.3	39	M	nuchal skin edema but without nuchal lymphatic cysts, brain autopsy brain un available	No
637	37	G1P0	abnormal shape of lateral ventricles, absent cavum septi pellucidi (suspected absence of corpus callosum)	22.4	22.4	F	absence of corpus callosum confirmed by MRI	No
648	29	NA	hydrocephalus	32.3	30.3	M	NA	No
653	29	NA	ventriculomegaly, subependymal cysts	36.6	33.6	F	NA	No
657	28	G2P0	holoprosencephaly	27.5	24.2	F	holoprosencephaly confirmed by MRI	No
663	24	G2P1	ventriculomegaly	33.4	33.4	F	NA	No

664	27	NA	aplasia of corpus callosum, holoprosencephaly	23.6	20.1	F	syndactyly, holoprosencephaly, presence of corpus callosum not available due to brain damage, ambiguous genitalia	Yes
667	29	G1P0	microcephaly	34.4	29.1	M	microcephaly	No
690	22	G1P0	intracranial hemorrhage, pelvic kidney, persistent left superior vena cava	31.1	30.1	M	myocardial thickening, persistent left superior vena cava, pelvic kidney, brain the autopsy unavailable	No
691	29	G4P0	exencephaly	17.6	17.1	M	exencephaly	No
693	33	G1P0	Chiari II malformation	26	22.2	F	Chiari II malformation	No
730	27	G5P1	hydrocephalus, absent radius, abdominal cystic masses	18.5	16.5	M	NA	No
733	31	G7P1	holoprosencephaly	19	19.2	F	holoprosencephaly	No
747	22	NA	holoprosencephaly, endocardial cushion defect, coarctation of aorta, single umbilical artery	14.2	13.6	F	holoprosencephaly, polydactyly of right hand, the autopsy of heart unavailable	No
758	24	G1P0	intracranial hemorrhage	36.4	38.4	M	NA	No
771	26	G2P0	ventriculomegaly, hyperechoic under nuchal skin	26.3	26	M	a nuchal mass of 4*3.5*1.5cm, autopsy of the brain unavailable	No
772	33	NA	partial absence of cerebellar vermis, persistent right umbilical vein	22.3	22.6	F	absence of cerebellar vermis	No
777	31	G1P0	hydrocephalus, single umbilical artery	23.2	23.4	M	hydrocephalus	No
783	27	G1P0	ventriculomegaly, single umbilical artery	30.2	28.3	F	brain autopsy unavailable, no other malformation found	No

784	28	G1P0	arachnoid cysts	34.3	34.4	F	no other malformation found; brain autopsy unavailable	No
786	37	G6P0	Chiari II malformation	23	22	M	Chiari II malformation	No
796	27	G1P0	ventriculomegaly, IUGR	39.4	34.1	F	surface of the brain appears smooth, lacking sulci and gyri	No
809	28	G1P0	ventriculomegaly	29.6	28	F	brain autopsy unavailable, no other malformation found	No
810	24	G1P0	nuchal lymphatic cysts, holoprosencephaly	14	12.3	F	nuchal lymphatic cysts, holoprosencephaly	No
814	27	NA	Dandy-Walker malformation	22.1	21.4	F	Dandy-Walker malformation	No
820	25	NA	hydrocephalus	30.2	30.5	F	hydrocephalus	No
827	40	NA	ventriculomegaly	19.5	19	F	brain autopsy unavailable, umbilical cord twist, no other malformation found	No
829	29	G1P0	exencephaly	12.3	11.4	M	exencephaly, absence of the anus	No
836	23	G1P0	hydrocephalus	26	28	F	hydrocephalus	No
837	27	NA	holoprosencephaly, arachnoid cysts, single umbilical artery	31.6	28	F	holoprosencephaly, arachnoid cysts with hemorrhage, single umbilical artery, double superior vena cava	Yes
857	28	G2P1	white matter lesions, porencephaly	31.4	32	M	white matter lesions, dilation of left lateral ventricle with hemorrhage confirmed by MRI,	No
864	32	G3P1	hydrocephalus, cerebellum hypoplasia, low conus medullaris	30.1	27.5	F	hemivertebrae of the third lumbar spine vertebra, low conus	Yes

							medullaris, right iliosacral joint malformation	
866	28	NA	exencephaly	14	13.3	M	exencephaly	No
871	28	G2P0	holoprosencephaly	18	17.6	M	holoprosencephaly	No
882	23	NA	nonechoic lesions in the cerebral midline	36.2	35.4	F	NA	No
884	32	G2P1	hypoplasia of corpus callosum, cyst at midline	32.3	32	M	hypoplasia of corpus callosum, cyst at midline	No
888	29	G1P0	exencephaly	16.4	13	M	exencephaly	No
889	25	NA	aplasia of corpus callosum	29.1	29.3	M	NA	No
901	33	G1P0	holoprosencephaly, endocardial cushion defect, coarctation of aorta	22.3	21.5	F	holoprosencephaly, endocardial cushion defect,coarctation of aorta	No
903	28	G1P0	aplasia of corpus callosum, polydactyly	36.2	34	F	polydactyly, brain autopsy unavailable	No
917	21	G1P0	hydrocephalus	32.2	31.1	F	brain autopsy unavailable	No
924	25	G1P0	aplasia of corpus callosum	31.3	31	F	aplasia of corpus callosum confirmed by MRI	No
931	23	G2P1	holoprosencephaly	13.1	13	F	holoprosencephaly	No
935	27	G3P0	holoprosencephaly	23	23	F	holoprosencephaly	No
938	23	G1P0	aplasia of corpus callosum, arachnoid cysts, VSD, bicuspid aortic valve	30.6	30.5	M	NA	No
942	31	NA	Dandy-Walker malformation	29.3	28.3	M	brain autopsy unavailable	No
947	30	G1P0	Chiari II malformation	18	18	M	Chiari II malformation	No
949	31	NA	spina bifida occulta, myelomeningocele	38.1	35.6	M	NA	No

954	23	G2P0	ventriculomegaly	29	26	M	NA	No
982	28	NA	hypoplasia of corpus callosum, abnormal shape of lateral ventricles	32.1	32.5	M	hypoplasia of corpus callosum, right lateral ventricle space-occupying lesion (MRI)	No
993	32	NA	hydrocephalus	27.2	26.4	F	brain autopsy unavailable	No
1001	23	NA	microcephaly, meningoencephalocele, cleft lip	11	15.2	M	microcephaly, meningoencephalocele, cleft lip	No
1004	31	NA	holoprosencephaly	23.5	23	M	holoprosencephaly	No
1008	27	NA	bilateral choroid plexus cysts	19.2	15.6	F	bilateral choroid plexus cysts confirmed by MRI, retroesophageal right subclavian artery	Yes
1022	24	NA	ventriculomegaly, VSD	23.1	23.1	F	NA	No
1057	29	G2P0	spina bifida	13.2	11.4	F	NA	No
1133	39	G3P1	Chiari II malformation	15	14.3	M	NA	No
1142	27	G1P0	increased NT, arachnoid cysts	14.1	13.5	M	NA	No
1279	26	NA	parietal meningocele, polydactyly, bilateral polycystic kidney	16.4	15.5	F	parietal meningocele, polydactyly, bilateral polycystic kidney	No
1447	26	NA	ventriculomegaly	31.3	29.2	F	NA	No

NA: Post-mortem examination was not available; F, female; M, male

Brain autopsy unavailable without other description means no apparent malformation of other part of the body was found, but the brain was not examined due to several reasons such as autopsy was not accepted by patients or the brain has been damaged during the pregnancy termination.

Supplementary Table 2 Candidate variants and Incident findings

Patient No.	Sonographic features	Gene	HGVSnom	Protein affected	Variant Type	Zygosity	Classification	Gene related diseases	Inheritance	Clinical significance
197	microcephaly	TUBGCP4	NM_001286414.2:c.1740_1741dup	His581Leufs*4	Frameshift	het	LP	microcephaly	AR	Carrier
197	microcephaly	PCNT	NM_006031.5:c.7691-1G>A	N. A	SpliceAcceptor	het	LP	microcephaly	AR	Carrier
693	Chiari II malformation	MYRF	NM_001127392.2:c.567_570del	Pro191Hisfs16	Frameshift	het	LP	Encephalitis/encephalopathy, mild, with reversible myelin vacuolization	AD	Incident
269	subependymal cysts	SELENON	NM_020451.2:c.1397G>A	Arg466Gln	Missense	het	P	Myopathy, congenital, with fiber-type disproportion	AD|AR	Incident
563	hydrocephalus	TNNI3	NM_000363.4:c.433C>T	Arg145Trp	Missense	het	P	Cardiomyopathy	AD|AR	Secondary
175	spina bifida	COL3A1	NM_000090.3:c.1744G>A	Gly582Ser	Missense	het	P/LP	Ehlers-Danlos syndrome	AD	Secondary
138	microcephaly, meningoencephalocele	ELN	NM_000501.3:c.2032+1G>A	N. A	SpliceDonor	het	LP	Cutis laxa, autosomal dominant 1	AD	Incident
175	spina bifida	LDLR	NM_000527.4:c.241C>T	Arg81Cys	Missense	het	LP	Hypercholesterolaemia	AD|AR	Secondary

het, heterozygous; homo, homozygous; hemi, hemizygous
P, pathogenic; LP, likely pathogenic; VUS, variant of uncertain significance
AD, autosome dominant; AR autosome recessive; XLD, X-linked dominant
N.A: not applicable

Supplementary Table 3 Planar cell polarity gene list

ANKRD6
CELSR1
CELSR2
CELSR3
DVL2
DVL3
FZD6
PRICKLE1
VANGL1
VANGL2
LRP6
PTK7
SCRIB

Supplementary Table 4 CNVs pathogenicity interpretation criteria

CNV	Size	Number of RefSeq genes	Number of OMIM genes	ClinGen CNV Interpretation Scoring			Syndromes	Reference
				Score	Evidence Type	Pathogenicity		
del(1p36.2p36.3).seq(823534-15632453)x1	14.8Mb	219	35	1.9	1A+2A+3C	P	1p36 deletion syndrome	(Wu et al., 1999)
dup(2q36.3q37.3).seq(226537458-242997727)x3	16.5Mb	168	30	1.9	1A+2A+3C	P		(Ma et al., 2015)
del(6q26q27).seq(161507517-170879606)x1	9.4Mb	52	6	1.9	1A+2A+3C	P	6q terminal deletions syndrome	(Elia et al., 2006)
del(3q12.1q21.2).seq(99540858-125130561)x1	25.5Mb	161	22	1.9	1A+2A+3C	P	3q13.31 deletion syndrome region	(Molin et al., 2012)
del(4q31.3q32.1).seq(153436540-160087839)x1	6.6Mb	41	10	1.9	1A+2A+3C	P		(Fabretto et al., 2012)
dup(5p14.3p15.3).seq(10429-23273021)x3	23.2Mb	90	14	1.9	1A+2A+3C	P		(Rethore et al., 1989)
del(18p11.3p11.3).seq(111935-4272634)x1	4.2Mb	27	3	1.45	1A+2A+3B	P		(Taniguchi, Anderson, Sutherland, & Wotton, 2012)
del(6q25.3q27).seq(160630268-170879606)x1	10.2Mb	57	8	1.9	1A+2A+3C	P		(Nagamani et al., 2009)
dup(7p22.1).seq(5029498-	1.8Mb	35	4	1.45	1A+2A+3B	P	7p22.1	(Caselli et

6809995) x3							microduplication syndrome	al., 2015)
del(7q35q36.3).seq(149299056-159068966)x1	9.8Mb	87	12	1.9	1A+2A+3C	P		(Horn et al., 2004)
del(7q33q36.3).seq(137529688-159068966)x1	21.5Mb	203	27	1.9	1A+2A+3B	P		
del(7q35q36.1).seq(145049787-159068966)x1	14.0Mb	104	14	1.9	1A+2A+3B	P		
dup(19q13.42q13.43).seq(55330867-59044235)x3	3.7Mb	139	6	0.9	1A+2B+3C+5F	LP		(Carvalheira et al., 2014)
del(8p11.21).seq(41835654-41836946)x1	1.3Kb	1	1	1.05	1A+2E+3A+4C+5F	P		(Arboleda et al., 2015)
dup(9p24.1p24.3).seq(10001-6476812)x3	6.5Mb	43	8	1.2	1A+2B+3B+4H+4M+5F	P		(Glessner et al., 2017)
del(13q22.1q34).seq(74369596-115054392)x1	40.7Mb	165	26	1.9	1A+2A+3C	P	13q deletion syndrome	(Quelin et al., 2009)
dup(13q31.2q34).seq(89863557-115054392)x3	25.2Mb	127	20	1.9	1A+2A+3C	P	partial 13q trisomy	(Rogers, 1984)
del(20p13).seq(60001-2226344)x1	2.2Mb	39	6	1.9	1A+2A+3C	P	20p13 microdeletion syndrome	(An et al., 2013)
del(17p13.2p13.3).seq(1081133-4774754) x1	3.7Mb	88	12	1.9	1A+2A+3C	P	17p13.3 deletion syndrome (Miller-Dieker syndrome)	(Bruno et al., 2010)

dup(17p13.3).seq(1150479-1592862) x3	442Kb	12	3	1	1A+2A+3A	P	17p13.3 microduplication syndrome	(Curry et al., 2013)
del(17q11.2).seq(29447084-29503935)x1	56.8Kb	1	1	1.65	1A+2E+3A+4A+4B+5F	P	NF1 microdeletion syndrome	(Imbard et al., 2015)
del(17q11.2).seq(228230706-228234866)x1‡	4.16Kb	1	1	0.9	1A+2E+3A+4H+5F	LP		(Wiszniewski et al., 2013)
del(18p11.2p11.3).seq(111935-15323954)x1	15.2Mb	87	11	1.9	1A+2A+3C	P		(Taniguchi et al., 2012)
del(18p11.2p11.3).seq(111935-15334797)x1	15.2Mb	87	11	1.9	1A+2A+3C	P		(Taniguchi et al., 2012)

Supplementary Table 5 SNVs pathogenicity interpretation criteria

Patient No.	Gene	HGVSnom	Affected protein	Variation	Zygosity	Classification	Related disease and inheritance	Interpretation criteria
49	KAT6B	NM_012330.3:c.3747delA	Gly1251Glufs*21	Frameshift	Het	P	Genitopatellar syndrome, AD	PVS1,PM2,PP4
432	STIL	NM_003035.2:c.3835C>T	Arg1279Cys	Missense	Het	LP	Microcephaly 7, primary, AR	PM2,PP1,PP3,PP4,BP1
	STIL	NM_003035.2:c.2344_2347delTTGC	Leu782Thrfs*2	Frameshift	Het	P		PVS1,PM2,PP4,PP1
211	CC2D2A	NM_001080522.2:c.3829T>C	Cys1277Arg	Missense	Het	LP		PM1,PM2,PP3,PP4

	CC2D2A	NM_001080522.2:c.3874G>T	Asp1292Tyr	Missense	Het	LP	Joubert syndrome 9, AR	PM1,PM2,PP3,PP4
234	KAT6B	NM_012330.3:c.3660dup	Arg1221*fs*1	Frameshift	Het	P	Genitopatellar syndrome, AD	PVS1,PM2,PP4
246	TUBA1A	NM_006009.3:c.748G>T	Val250Phe	Missense	Het	LP	Lissencephaly 3, AD	PM1,PM2,PP2,PP3
273	GJC2	NM_020435.3:c.1125_1135delCGGCCTCCCTG	Ala379Glyfs*109	Frameshift	Het	LP	Lymphatic malformation 3, AD	PVS1,PM2
348	DNM1L	NM_012062.4:c.345_346delAG	Glu116Lysfs*6	Frameshift	Het	LP	Encephalopathy, AD	PVS1,PM2
357	FGFR3	NM_000142.4:c.1948A>G	Lys650Glu	Missense	Het	P	Thanatophoric dysplasia, AD	PS1, PM1,PM2,PP3,PP4
431	TUBA1A	NM_006009.3:c.614A>T	Asp205Val	Missense	Het	LP	Lissencephaly 3, AD	PM1,PM2,PP2,PP3
464	PDHA1	NM_000284.3:c.923_929delAGGAAGT	Ser312Valfs*12	Frameshift	Het	LP	Pyruvate dehydrogenase E1α deficiency, XLD	PVS1,PM2
479	GLI2	NM_005270.4:c.94dup	Ala32Glyfs*34	Frameshift	Het	LP	Culler-Jones syndrome Holoprosencephaly 9, AD	PVS1,PM2

558	PTPN11	NM_002834.4:c.1403C>T	Thr468Met	Missense	Het	P	Noonan syndrome 1, AD	PS1,PS3,PM1,PP3, PP5
627	NSD1	NM_022455.4:c.5177C>T	Pro1726Leu	Missense	Het	LP	Sotos syndrome, AD	PS1,PM1,PM2,PP3
648	FOXG1	NM_005249.4:c.171_180delCC CGCCGCCG	Pro60Argfs*129	Frameshift	Het	LP	Rett syndrome, congenital variant, AD	PVS1,PM2
730	FANCC	NM_000136.2:c.1330-1G>A	N.A	Splice	Homo	LP	Fanconi anemia, complementat ion group C, AR	PVS1,PM2
733	SIX3	NM_005413.3:c.339G>A	Trp113*	Nonsense	Het	P	Holoprosence phaly 2, AD	PVS1,PS1,PS3,PM2
836	SOX2	NM_003106.3:c.480C>G	Tyr160*	Nonsense	Het	P	Optic nerve hypoplasia and abnormalities of the central nervous system, AD	PVS1,PS1,PM2
903	OFD1	NM_003611.2:c.1103_1106delT GAT	Ile369Lysfs*18	Frameshift	Het	LP	Orofaciodigital syndrome I, XLD	PVS1,PM2

924	ARX	NM_139058.2:c.1074-1G>A	N.A	Splice	Het	LP	Lissencephaly, XL2	PVS1,PM2
942	NID1	NM_002508.2:c.1786C>T	Arg596*	Nonsense	Het	LP	Dandy-Walker malformation, AD	PVS1,PM2
993	PDHA1	NM_000284.3:c.933_936delAAGT	Ser312Argfs*13	Frameshift	Het	P	Pyruvate dehydrogenase E1α deficiency, XLD	PVS1,PS1,PM2
1278	CC2D2A	NM_001080522.2:c.4333C>T	Arg1445*	Nonsense	Homo	P	Meckel syndrome 6, AR	PVS1,PM2,PP4
222	PTCH1	NM_000264.3:c.2757_2758delCT	Phe919Leufs*39	Frameshift	Het	LP	Basal cell nevus syndrome, AD	PVS1,PM2
114	NF1	NM_001128147.2:c.1742dup	Leu581Phefs*6	Frameshift	Het	P	Neurofibromatosis, type I, AD	PVS1,PP3,PP5

Het, heterozygous; Homo, homozygous;
P, pathogenic; LP, likely pathogenic
AD, autosome dominant; AR autosome recessive; XLD, X-linked dominant
N.A: not applicable

Supplementary Table 6 Primer List

Patient No.	Gene	HGVSnom	F	R
49	KAT6B	NM_012330.3:c.3747delA	TACTAAAGAAGAAAAGGGGTCG	AGCTTGTAAGGATGGGAAAG
432	STIL	NM_003035.2:c.3835C>T	GGGACCAACGCAGATACGCCAGT	GTCCTATCACCAGCCAGCCATCTCAC
	STIL	NM_003035.2:c.2344_2347delTTGC	TCTGATGCCCTGTTCCCCTA	GGTTTTGGTCCTACTGCACC
211	CC2D2A	NM_001080522.2:c.3829T>C	GGGACACCAGGACATGACAG	CCTGGCTGCCTTCAGACTTT
	CC2D2A	NM_001080522.2:c.3874G>T		
234	KAT6B	NM_012330.3:c.3660dup	TACTAAAGAAGAAAAGGGGTCG	AGCTTGTAAGGATGGGAAAG
246	TUBA1A	NM_006009.3:c.748G>T	ACAGGCAGCAAGCCATGTAT	CAACTCCATCCTCACCACCC
273	GJC2	NM_020435.3:c.1125_1135delCGGCCTCCCTG	CCTGCTGCTCAACCTCTGT	CGGCTAAGGAGAAGGCTGAG
348	DNM1L	NM_012062.4:c.345_346delAG	CCTTGAAGTATATGGACTCCCCC	GTGAAACAATGCTTCCTGAGTTAG
357	FGFR3	NM_000142.4:c.1948A>G	TCACTGGCGTTACTGACTGCG	CGGGAGATCTTGTGCACGGT
431	TUBA1A	NM_006009.3:c.614A>T	ATCAAATCTCAGGGAAGCAG	CCTGGAAGATGTATGAAAAGTG
464	PDHA1	NM_000284.3:c.923_929delAGGAAGT	GAAGCAGTTATTACAGAATGTTAGG	TCACTTTCTGGGCTTGGAG
479	GLI2	NM_005270.4:c.94dup	TTGTCCGTGAGGTCGTTCAG	CCTTCATGGTGCCTGGGATT
558	PTPN11	NM_002834.4:c.1403C>T	CCCGGCCCTCTTTCAGTAAT	ACGAAGTGGCAGAAGTCAGG
627	NSD1	NM_022455.4:c.5177C>T	CCAACCTGAACCTGACGAGAGTTAA	GAAGAAGAGGGGAGGAGTACCATGA
648	FOXG1	NM_005249.4:c.171_180delCCCGCCGCCG	AGGAGGAGGAGGAGGAGGAA	GTTGATGCTGAACGAGGACTTG
730	FANCC	NM_000136.2:c.1330-1G>A	AGTCAAGAAATTTCCATTCCGCCC	GAGTACTAGGATGCTGGACCACAG

733	SIX3	NM_005413.3:c.339G>A	GATTCTCACCACCGCTCCATACT	TTGAAGCAATGCGTCTTCTGCTC
836	SOX2	NM_003106.3:c.480C>G	CGTTCATCGACGAGGCTAAG	TGCGAGTAGGACATGCTGTAG
903	OFD1	NM_003611.2:c.1103_1106delTGAT	CAGTTTATAGGATCAAGGAAGG	GACCCAACTTGGGAAGC
924	ARX	NM_139058.2:c.1074-1G>A	TTGAAGCCACCCTTGGTTGT	ACTGCAGTCTCAGGGACTCT
942	NID1	NM_002508.2:c.1786C>T	CTGTCATCTGGTAGCCTTATGG	CTTCTACAGACTGAGAGTAAGAGAC
993	PDHA1	NM_000284.3:c.933_936delAAGT	GAAGCAGTTATTACAGAATGTTAGG	TCACTTTCTGGGCTTGGAG
1278	CC2D2A	NM_001080522.2:c.4333C>T	ACACTTGCTGTCTCTCT	ATCAAAACTTTTTCTTC
222	PTCH1	NM_000264.3:c.2757_2758delCT	GCCAGTGATTGCATCCTCCGATA	CAGGGAAGGCACCTCTGTAAGTT
114	NF1	NM_001128147.2:c.1742dup	GACTTTGTGGCAAGTGAGACAG	AAAACCATAAAACCTTTGGAAGTGT
653	KAT6A(EX6)	del(8p11.21).seq(41835654-41836946)x1	AGTTGGAATCTCACTCTGTTGC	GAGGTTGACAAGCCTGAAGAC
884	NF1(EX2-EX5)	del(17q11.2).seq(29447084-29503935)x1	TGAGGTATCTGTTCTGTTCCATTG	CCAGCCAGAGAGGACACAAA
954	TM4SF20(EX3)	del(17q11.2).seq(228230706-228234866)x1	CCAGGAGGCAGAGGTTACAG	TCTCTTCACTCTCATAAAGGGAATC
			CTCAGTCTTCCGTGGATTGTC	TGTTCTCTTCACTCTCATAAAGGG
