# Supplementary material for: Genomic architecture of fetal central nervous system anomalies using whole-genome sequencing
Source: NPJ Genom Med. 2022 May 13;7:31. doi: 10.1038/s41525-022-00301-4 (PMC9106651; doi:10.1038/s41525-022-00301-4)
Supplement: Supplementary file 2 — Reporting Summary Checklist [file 41525_2022_301_MOESM2_ESM.pdf]

nature research

Corresponding author(s): Xinlin Chen NPJGENMED-00711R2

Last updated by author(s): Mar 24, 2022

Reporting Summary

Nature Research wishes to improve the reproducibility of the work that we publish. This form provides structure for consistency and transparency in reporting. For further information on Nature Research policies, see our Editorial Policies and the Editorial Policy Checklist.

Statistics

For all statistical analyses, confirm that the following items are present in the figure legend, table legend, main text, or Methods section.

n/a	Confirmed	
☐	☒	The exact sample size (*n*) for each experimental group/condition, given as a discrete number and unit of measurement
☒	☐	A statement on whether measurements were taken from distinct samples or whether the same sample was measured repeatedly
☐	☒	The statistical test(s) used AND whether they are one- or two-sided<br>*Only common tests should be described solely by name; describe more complex techniques in the Methods section.*
☒	☐	A description of all covariates tested
☒	☐	A description of any assumptions or corrections, such as tests of normality and adjustment for multiple comparisons
☐	☒	A full description of the statistical parameters including central tendency (e.g. means) or other basic estimates (e.g. regression coefficient) AND variation (e.g. standard deviation) or associated estimates of uncertainty (e.g. confidence intervals)
☐	☒	For null hypothesis testing, the test statistic (e.g. *F*, *t*, *r*) with confidence intervals, effect sizes, degrees of freedom and *P* value noted<br>*Give P values as exact values whenever suitable.*
☒	☐	For Bayesian analysis, information on the choice of priors and Markov chain Monte Carlo settings
☒	☐	For hierarchical and complex designs, identification of the appropriate level for tests and full reporting of outcomes
☒	☐	Estimates of effect sizes (e.g. Cohen's *d*, Pearson's *r*), indicating how they were calculated

Our web collection on statistics for biologists contains articles on many of the points above.

Software and code

Policy information about availability of computer code

Data collection	data in this study were collected using Edico Genome v2.4.1, SpeedSeq SV pipeline (v.0.0.3a). bcfanno (v1.4).
Data analysis	R version 3.5.2, Python 3.6. Custom R and Python scripts used for the singleton variant analyses are available upon request.

For manuscripts utilizing custom algorithms or software that are central to the research but not yet described in published literature, software must be made available to editors and reviewers. We strongly encourage code deposition in a community repository (e.g. GitHub). See the Nature Research guidelines for submitting code & software for further information.

Data

Policy information about availability of data

All manuscripts must include a data availability statement. This statement should provide the following information, where applicable:

- Accession codes, unique identifiers, or web links for publicly available datasets
- A list of figures that have associated raw data
- A description of any restrictions on data availability

Project information is uploaded to NCBI with accession number PRJNA798107. Data are uploaded to NCBI dbSNP with the web link https://www.ncbi.nlm.nih.gov/SNP/snp_viewTable.cgi?handle=YY_MCH. Data are also publicly available in the China National GeneBank Database (CNGB) Nucleotide Sequence Archive (Accession: CNP0002431).

Field-specific reporting

Please select the one below that is the best fit for your research. If you are not sure, read the appropriate sections before making your selection.

☒ Life sciences ☐ Behavioural & social sciences ☐ Ecological, evolutionary & environmental sciences

For a reference copy of the document with all sections, see nature.com/documents/nr-reporting-summary-flat.pdf

Life sciences study design

All studies must disclose on these points even when the disclosure is negative.

Sample size	No sample size calculation. All available samples that fulfill criteria as described in Materials and Methods were included.
Data exclusions	small CNVs in four samples have been excluded due to low quality of variants identification
Replication	Replication has not been done in this study. This study focused on primary diagnosis of fetal anomalies. Diagnostic variants mainly consist of extremely rare variants and de novo mutations.
Randomization	In rare singleton deleterious variants analysis, the case group consisted of the 136 samples in the cohort where deep WGS data were available and the control group consisted of 200 randomly selected individuals from the in-house population database.
Blinding	This study focused on displaying the genetic architecture and identifying primary diagnosis of fetal anomalies. No treatment or intervention were involved. Therefore, blinding is irrelevant.

Reporting for specific materials, systems and methods

We require information from authors about some types of materials, experimental systems and methods used in many studies. Here, indicate whether each material, system or method listed is relevant to your study. If you are not sure if a list item applies to your research, read the appropriate section before selecting a response.

Materials & experimental systems

n/a	Involved in the study	
☒	☐	Antibodies
☒	☐	Eukaryotic cell lines
☒	☐	Palaeontology and archaeology
☒	☐	Animals and other organisms
☐	☒	Human research participants
☐	☒	Clinical data
☒	☐	Dual use research of concern

Methods

n/a	Involved in the study	
☒	☐	ChIP-seq
☒	☐	Flow cytometry
☒	☐	MRI-based neuroimaging

Human research participants

Policy information about studies involving human research participants

Population characteristics	Participants are patients whose fetus were found structure anomalies in-uterus during prenatal ultrasound scan. They are all Chinese woman, and most of them are from Hubei Provence China. The median maternal age of the cohort was 27.
Recruitment	Patients were found by a prenatal ultrasound screening in a multiple-level referral system of prenatal screening and diagnosis between 2015-2017 were retrospectively reviewed. This referral system was launched and conducted by Maternal and Child Health Hospital of Hubei Province (MCHH), which includes 156 prenatal screening units and 32 diagnostic centers all over Hubei Provence as well as several districts in middle and southwestern China. All of the screening units and diagnostic centers were trained by MCHH followed practice guideline laughed by The International Society of Ultrasound in Obstetrics & Gynecology(ISUOG)12. If fetal structural anomalies were detected during a routine ultrasound scan at any these units and diagnostic centers, Then patients were transferred to MCHH for thorough systematic or special prenatal ultrasound by at least two chief physicians to get a final diagnosis. MRI was also provided when it was accepted by patients and their families. Patients who opted for donating invasive testing samples, abortion samples or birth samples were invited participation in a congenital defect study. For this study, patients diagnosed with fetal CNS anomalies were identified form the congenital defect study by reviewing the medical records and images.
Ethics oversight	This project followed Chinese state and local regulations related to biological and medical researches and was approved by Maternal and Child Health Hospital of Hubei Provinceand BGI Ethics Committee

Note that full information on the approval of the study protocol must also be provided in the manuscript.

Clinical data

Policy information about clinical studies

All manuscripts should comply with the ICMJE guidelines for publication of clinical research and a completed CONSORT checklist must be included with all submissions.

Clinical trial registration	*Provide the trial registration number from ClinicalTrials.gov or an equivalent agency.*
Study protocol	*Note where the full trial protocol can be accessed OR if not available, explain why.*
Data collection	*Describe the settings and locales of data collection, noting the time periods of recruitment and data collection.*
Outcomes	*Describe how you pre-defined primary and secondary outcome measures and how you assessed these measures.*
